# HIV-1 Env-Specific Memory and Germinal Center B Cells in C57BL/6 Mice

**DOI:** 10.3390/v6093400

**Published:** 2014-09-05

**Authors:** Martina Soldemo, Gabriel K. Pedersen, Gunilla B. Karlsson Hedestam

**Affiliations:** Department of Microbiology, Tumor and Cell Biology, Karolinska Institutet, S-171 77 Stockholm, Sweden; E-Mails: Martina.Soldemo@ki.se (M.S.); Gabriel.Pedersen@ki.se (G.K.P.)

**Keywords:** B cells, antibody, immunization, germinal center, HIV-1, envelope glycoprotein, BALB/c and C57BL/6 mice

## Abstract

Continued efforts to define the immunogenic properties of the HIV-1 envelope glycoproteins (Env) are needed to elicit effective antibody (Ab) responses by vaccination. HIV-1 is a highly neutralization-resistant virus due to conformational and glycan shielding of conserved Ab determinants on the virus spike. Elicitation of broadly neutralizing Abs that bind poorly accessible epitope regions on Env is therefore extremely challenging and will likely require selective targeting of specific sub-determinants. To evaluate such approaches there is a pressing need for *in vivo* studies in both large and small animals, including mice. Currently, most mouse immunization studies are performed in the BALB/c strain; however, the C57BL/6 strain offers improved possibilities for mechanistic studies due to the availability of numerous knock-out strains on this genetic background. Here, we compared Env immunogenicity in BALB/c and C57BL/6 mice and found that the magnitude of the antigen-specific response was somewhat lower in C57BL/6 than in BALB/c mice by ELISA but not significantly different by B cell ELISpot measurements. We then established protocols for the isolation of single Env-specific memory B cells and germinal center (GC) B cells from immunized C57BL/6 mice to facilitate future studies of the elicited response at the monoclonal Ab level. We propose that these protocols can be used to gain an improved understanding of the early recruitment of Env-specific B cells to the GC as well as the archiving of such responses in the memory B cell pool following immunization.

## 1. Introduction

Mouse models have been, and remain, central for our understanding of basic immunology of vaccine-induced immune responses. The ease by which mice can be genetically manipulated combined with the availability of comprehensive genetic data bases, short breeding cycles and relatively low costs make mice a superior animal model for questions of basic and mechanistic nature. Our current understanding of lymphocyte development and host responses to antigen stimulation comes largely from studies in mice. Despite this, mice are relatively under-used in HIV-1 vaccine research, in part because they are naturally resistant to HIV-1 infection preventing their use in experiments requiring virus challenge. This limitation has been addressed by generating mouse models that support HIV-1 replication [1] or HIV-1 entry [2]. For example, successful HIV-1 replication was achieved in humanized mice generated by engraftment of CD34^+^ hematopoetic cells into Rag2^−/−^γc^−/−^ mice (human immune system, HIS) mice [3]. Thus far, this model cannot be used for vaccine evaluation due to insufficient development of immune functions after engraftment of human cells, but improvements to the model are underway suggesting that some of the current challenges hampering its use for vaccine studies may eventually be overcome [4,5].

Challenge studies are, however, not generally required in the first phases of vaccine testing. For HIV-1 envelope glycoprotein (Env)-based vaccine candidates aimed to elicit broadly neutralizing antibodies (bNAbs), neutralization breadth of elicited serum responses is best evaluated in standardized *in vitro* assays using panels of genetically diverse single-cycle infectious viruses [6]. The choice of animal model used for such studies is usually determined based on practical criteria such as animal availability, volume of sera that can be obtained following vaccine inoculation and cost. In this regard, rabbits and guinea pigs are well-established models for serological studies and are often preferred over mice since larger volumes of sera can be collected. However, rabbits and guinea pigs are not amenable to detailed immunological investigations due to the limited number of reagents available for cellular analysis and incomplete genetic information limiting their use for detailed immunological analysis. Instead, non-human primates (NHPs), notably rhesus macaques, have emerged as an interesting alternative model for analyses of vaccine-induced responses since large volumes of sera can be collected, reagents for cellular analyses are available and they are genetically highly homologous to humans, meeting several important practical criteria for a useful animal model. Over the past years, we have established methodology and systems for high-resolution analysis of vaccine-induced B cell responses in NHPs to extend this model beyond its use as a challenge model [7,8,9,10,11]. Using these protocols, we investigated vaccine-induced memory B cell and plasma cell frequencies in blood and bone marrow, as well as genetic properties of Abs such as gene segment use, clonality and level of somatic hypermutation (SHM) of Env-specific Abs. The NHP model has direct translational value for our understanding of vaccine-induced responses in humans. However, for ethical, practical and cost reasons the number of studies that can be performed in NHPs is limited and small animal models remain critical for most basic research questions.

Thus far, relatively few studies have exploited available mouse models for detailed investigation of B cell responses to HIV-1 Env, from the initial activation of naïve B cells to the establishment of Env-specific memory B cells or plasma cells. In contrast, there is an extensive literature from studies in mice using non-pathogen-derived antigens, such as hen egg lysozyme (HEL) and the hapten-carrier antigen NP-CGG, several which were performed in mice transgenic for antigen-specific B cell receptors [12,13,14,15,16]. These studies have laid the foundation for our current understanding of humoral immunity. The application of similar experimental approaches to studies of real-world vaccine antigens such as viral glycoproteins is therefore of significant interest. The recent development of transgenic mice expressing human HIV-1 bNAbs isolated from chronically infected individuals provides new and exciting opportunities for basic investigations of the development of Env-specific B cell responses following immunization [17,18].

Studies in chronically HIV-1 infected individuals highlight that extensive affinity maturation through SHM is required for the development of bNAbs [19]. The extent to which different vaccine modalities promote SHM of Abs recognizing distinct sub-determinants on Env, including bNAb epitopes, is not well understood and is a question suitable for studies in mice. We previously showed that inoculation of BALB/c mice with recombinant, soluble HIV-1 Env trimers (gp140-F) administered in adjuvant, stimulated robust Ab and memory B cell responses [20,21]. Here, we set out to compare Env immunogenicity in BALB/c and C57BL/6 mice and to establish a protocol for the detection of Env-specific memory and GC B cells in C57BL/6 mice. We propose that the protocols described here can be used for future studies of HIV-1 Env-elicited vaccine responses to investigate elicited Ab sub‑specificities and B cell selection at the single cell level in a variety of mouse strains on the C57BL/6 background.

## 2. Materials and Methods

*Recombinant HIV-1 Env glycoproteins.* Recombinant soluble Env gp140 trimers (gp140-F) based on the YU2 isolate of HIV-1 were used for immunizations [22]. Biotinlylated gp140-F trimers were used as probes in the B cell ELISpot assay, as previously described [20], and for detection of Env‑specific cells by flow cytometry. Briefly, HEK 293F cells in FreeStyle 293 Expression medium (Invitrogen, Carlsbad, CA, USA) at a cell density of 1.2 × 10^6^ cells/mL, were transient DNA transfected with 293Fectin (Invitrogen) and OPTI-MEMI medium (Invitrogen). The transfection was harvested at day 5 after transfection by centrifugation at 3500 × *g* to remove cells and cell debris. Supernatant where filtered through a 0.22 µM filter unit and supplemented with 100 U/mL penicillin and 100 µM streptomycin and complete EDTA-free protease inhibitor cocktail (Roche, Mannheim, Germany). Proteins were then purified in a two-step chromatography purification process. First, proteins were captured in a lentil-lectin affinity chromatography (GE Healthcare, Uppsala, Sweden) via the glycans and after extensive washing in PBS/0.5M NaCl, the proteins were eluted in elution buffer (1 M methyl-α-_D_-mannopyranoside, 10 mM imidazole, PBS and 0.5 M NaCl). Second, eluted proteins were then captured in a nickel-chelating chromatography (GE Healthcare) via the His-tag. The column was extensively washed in washing buffer (40 mM imidazole, 200 mM sodium phosphate buffer and 0.5 M NaCl) before elution in elution buffer (300 mM imidazole, 200 mM sodium phosphate buffer and 0.5 M NaCl). Protein elutes were concentrated with an Amicon Ultra 30 kDa cut-off concentrator (Millipore, Darmstadt, Germany). Probes for ELISpot and FACS were site‑specific biotinylated at the Avitag sequence by using Biotin-protein ligase biotinylation kit (GeneCopoeia, Rockville, MD, USA) according to manufacturer’s instruction. Env gp140-F trimers are from here referred to as Env.

*Animals, vaccine inoculations and preparation of single cells.* Adult male BALB/c and C57BL/6 mice, obtained originally from Jackson Laboratory, and bred at the animal facility, MTC, Karolinska Institutet, were immunized at age of 6–9 weeks with 10 µg Env together with 10 µg of AbISCO-100 adjuvant (Isconova, Uppsala, Sweden) or Imject^®^Alum (Thermo Scientific/Pierce, Rockford, IL, USA). Mice were inoculated one, two or three times either by sub-cutaneous (s.c.) or intraperitoneal (i.p.) injections. All animal experiments were approved by the Committee for Animal Ethics (Stockholm, Sweden) and performed according to given guidelines. The mice were sacrificed by cervical dislocation and cells from inguinal lymph nodes (ingLN) and spleens were prepared in single cell suspension by passing the tissues through a 70 µM nylon cell strainer. Red blood cells were lysed with hypotonic ammonium chloride solution and the remaining cells were resuspended in complete RPMI 1640 medium containing 5% FCS, 50 µM 2-ME, 2 mM L-glutamine, 100 U/mL penicillin and 100 µM streptomycin.

*ELISA for serological antibody responses.* To detect total Env-specific or isotype Env-specific antibody responses 96-well ELISA plates (Nunc, Roskilde, Denmark) were pre-coated with 100 ng/well (1 µg/mL) of *Galanthus nivalis* lectin (Sigma-Aldrich, Saint Louis, MO, USA) diluted in PBS and incubated overnight (ON) at 4 °C. Plates were washed in washing buffer (PBS containing 0.05% Tween-20) to remove excess lectin before addition of 100 ng/well (1 µg/mL) of Env protein diluted in PBS. Plates were incubated for 2 hours (h) at room temperature (RT) before plates were washed in washing buffer and blocked in blocking buffer (PBS containing 2% nonfat dry milk) at RT for 1 h. Blocking buffer was removed and serum from immunized mice was added in serial dilutions in blocking buffer and incubated at RT for 2 h. After washing the plates in washing buffer, secondary antibodies were added; goat anti-mouse IgG‑horse radish peroxidase (HRP) (Southern Biotech, Birmingham, AL, USA) diluted 1:1000, goat anti-mouse IgG1-HRP (Southern Biotech) diluted 1:5000, goat anti-mouse IgG2a-HRP (Southern Biotech) diluted 1:5000, goat anti-mouse IgG2b-HRP (Southern Biotech) diluted 1:5000, goat anti-mouse IgG2c-HRP (Southern Biotech) diluted 1:5000 or goat anti-mouse IgG3-HRP (Southern Biotech) diluted 1:1500. All secondary HRP-conjugated antibodies were diluted in washing buffer and 100 µL was added to each well and incubated at RT for 1 h. Plates were washed in washing buffer before they were developed. To develop the plates, 100 µL of TMB Stabilized Chromogen substrate (Invitrogen) was added and incubated for 10 min in RT. The reaction was stopped by addition of 100 µL 1 M H_2_SO_4_ to the wells and the optical density (OD) was measured at 450 nm using an Asys Expert 96 ELISA reader (Biochrom, Cambridge, UK).

*B cell ELISpot assay.* Total IgG and Env-specific antibody secreting cells (ASCs) were enumerated in a B cell ELISpot assay, as previously described [20]. Briefly, 96-well Multiscreen-IP filter plates (Millipore) were pre-treated with 70% ethanol and washed three times in PBS followed by coating with 1 µg/well (10 µg/mL) of a polyclonal goat anti-mouse IgG antibody (Mabtech, Nacka Strand, Sweden). The plates were incubated ON at 4 °C. Excess coating antibody was removed and plates were washed five times in PBS and blocked in complete RPMI 1640 medium for 2 h at 37 °C before cells were added. Medium was removed from the plates and splenocytes in single cell suspension were added in duplicates to the wells in 3-fold dilutions starting at 10^6^ cells/well. The plates were wrapped in plastic and incubated for 12 h at 37 °C. To detect spots, cells were first removed and plates washed six times in washing buffer (PBS containing 0.05% Tween-20). For total IgG-secreting cells, a biotinylated polyclonal goat anti‑mouse IgG (Mabtech) diluted in blocking buffer (PBS containing 0.05% Tween-20 and 1% FCS) was added at a concentration of 100 ng/well (1 µg/mL) to each well. For detection of Env-specific ASCs, 200 ng/well (2 µg/mL) of biotinylated protein diluted in blocking buffer was added to the wells. Biotinylated proteins and antibodies were incubated in the plates for 2 h at RT. The plates were then washed six times in PBS and 100 µL streptavidin-ALP (Mabtech) diluted 1:1000 in PBS was added to the wells and incubated in RT for 45 min. To develop the spots, plates were washed six times in water and 100 µL of BCIP/NBT plus substrate (Mabtech) was added to wells and incubated for 10 min in RT. The reaction was stopped by emptying the plates and washing them extensively in water followed by air-drying. The spots were counted in an ImmunoSpot analyzer (CTL Immunospot, Shaker Heights, OH, USA).

*Virus neutralization assays*. Neutralization assays were kindly performed by the laboratory of John Mascola at the Vaccine Research Center at the NIH using a single round of infection HIV-1 Env pseudovirus assay and TZM-bl target cells [6]. The results are reported as the serum neutralization ID_50_, which is the reciprocal of the serum dilution producing 50% virus neutralization. Diverse HIV-1 virus isolates, including viruses from clades A, B and C were used in the neutralization assays. The sources of the Env-encoding plasmids were described previously [7].

*Flow cytometry analysis.* For detection of Env-specific memory B cells, samples were first incubated with Fc receptor block antibody (anti-CD16/32; BD Biosciences, San Diego, CA, USA) followed by addition of biotinylated Env protein pre-coupled to APC-conjugated streptavidin (Invitrogen). The following antibody panel was used for detection of memory B cells: APC-eFluor780 anti-B220 (RA3-6B2; eBioscience, San Diego, CA, USA), FITC anti-IgD (11-26c; eBioscience), FITC anti-IgM (polyclonal; Southern Biotech) PE-Cy7 anti-CD38 (Biolegend, San Diego, CA, USA). For detection of Env-specific GC B cells the samples were first incubated with Live/Dead AmCyan (93) followed by addition of biotinylated Env protein for all samples except control samples. The following antibody panel was used for detection of GC B cells: Pacific Blue anti-B220 (RA3-6B2), FITC anti-GL7 (GL7) and PerCP-Cy5.5 anti-IgD (11-26c.2a). APC-conjugated streptavidin (Invitrogen) was added to visualize biotinylated Env proteins. All antibodies come from BioLegend except Live/dead AmCyan (Invitrogen). Stained cells were fixed in fixation buffer (BD Bioscience) and analyzed on a MoFlo™ XDP (Beckman Coulter, Brea, CA, USA) or LSRFortessa™ cytometer (BD Biosciences, San Diego, CA, USA). Flow cytometry data were analyzed with Flowjo [23].

## 3. Results

### 3.1. Serological Assessment of Env-Specific Responses in BALB/c and C57BL/6 Mice

Mouse models remain critical for investigations of the evolution of B cell responses following vaccination. In the HIV-1 vaccine field, mice have not been considered an optimal host for the evaluation of Env-specific Abs due to the limited volumes of blood that can be collected for serological analysis as well as issues with non-specific background in HIV-1 neutralizing Ab assays. There is also concern that the relatively short immunoglobulin (Ig) D segments encoded by the mouse heavy chain Ig locus results in Abs possessing shorter HCDR3s compared to in primates and rabbits [24,25], which may hinder the development of antibodies targeting recessed epitopes on Env, such as the highly conserved CD4 binding site (CD4bs). However, antibody responses capable of neutralizing Tier 1 isolates of HIV-1 can be induced in mice [20,21,26,27], while Abs capable of neutralizing Tier 2 viruses were so far not elicited in any animal model. This suggests that the inability to elicit bNAbs observed so far is not restricted any given animal model.

**Figure 1:**
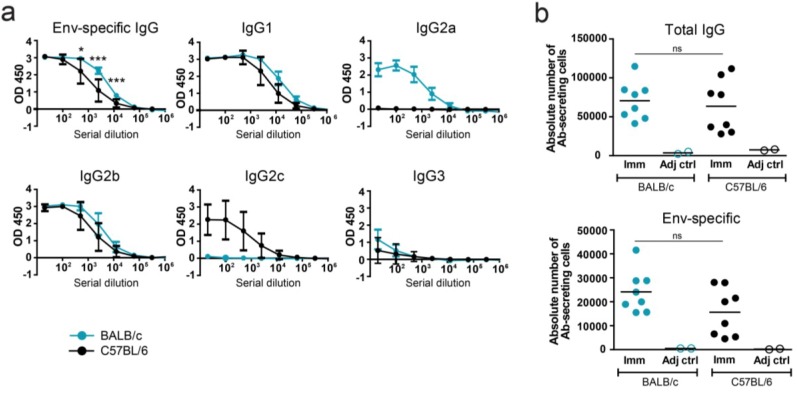
Antibody titers and numbers of antibody-secreting cells in BALB/c and C57BL/6 mice five days after two s.c. inoculations with Env in AbISCO-100. (**a**) Titers of Env-specific IgG and subclasses (IgG1, IgG2a, IgG2b, IgG2c and IgG3) at five-fold dilutions, starting at 1:20. Mean values and SD are shown in all diagrams. (**b**) Absolute numbers of total IgG and Env (gp140-F)-specific ASCs are shown for BALB/c (blue) and C57BL/6 (black) mice. The data shown are from one experiment with eight immunized mice/group and two adjuvant control mice/group. * and *** indicate statistically significant differences between BALB/c and C57BL/6 Env-specific IgG responses at the indicated dilutions, *p* < 0.05 and 0.001, respectively (Student’s t-test).

In prior HIV-1 Env immunization studies, BALB/c mice were most commonly used; however, for mechanistic studies, genetically engineered mice on the C57BL/6 background are often required. We therefore compared Env-elicited B cell responses in C57BL/6 and BALB/c mice as a starting point for future studies in the more versatile C57BL/6 strain. We first compared the overall Ab titers to soluble HIV-1 Env (gp140-F) trimers induced by prime-boosting BALB/c or C57BL/6 mice (n = 8 per strain) using a 14 day interval between the immunizations (Figure 1a). The purified Env was administered s.c. in the saponin-based adjuvant AbISCO-100 and Ab titers were measured by ELISA, five days following boosting. The total serum IgG responses were similar in BALB/c and C57BL/6 mice and were slightly increased in Env-immunized mice compared to in adjuvant control immunized mice but there was no measurable difference between the two strains (data not shown). In contrast, the Env-specific IgG response was significantly higher in BALB/c mice compared to C57BL/6 mice (*p* < 0.001, Student’s t-test) (Figure 1a). Two control mice from each strain immunized with adjuvant alone gave no detectable Env-specific IgG responses. When individual Ab isotypes were measured we found that the Env-specific IgG1 response was also somewhat higher in BALB/c compared to C57BL/6 mice and the same pattern was observed for IgG2b (Figure 1a). For Env-specific IgG2a and IgG2c, there was an inverse relation with high IgG2a produced in BALB/c mice but not in C57BL/6 mice and Env-specific IgG2c produced in C57BL/6 but not in BALB/c mice (Figure 1a), consistent with the respective IgG isotype expression of the two strains [28,29,30]. The Env-specific IgG3 response was very low in both mouse strains. Overall, IgG1 and IgG2b contributed strongest to the total Env-specific IgG response with contribution from either IgG2a or IgG2c, depending on the mouse strain used.

For a more quantitative measurement of Env-specific antibody-secreting cells (ASC) we next used a previously reported highly sensitive B cell ELISpot format [20]. Spleens from mice (n = 8) immunized twice with purified Env in adjuvant, or with adjuvant alone as a negative control (n = 2), were harvested five days after the second immunization and total IgG or Env-specific ASC were measured. In brief, Ab-secreting cells were quantified by incubation of splenocytes on plates coated with anti-IgG. Cells were washed away and the spots were detected with biotinylated anti-IgG or biotinylated Env. The time point chosen for analysis (five days after boost) was based on previous kinetic studies where we found that there is a peak in the plasma cell formation at this time, most likely resulting from proliferation and differentiation of antigen-specific memory B cells into short-lived plasma cells by this time point. The total numbers of IgG ASCs were similar between BALB/c and C57BL/6 mice (Figure 1b, upper panel) When Env-specific (total gp140-F-specific) ASCs were measured we observed a higher absolute number of antigen-specific cells in BALB/c (mean 2.4 × 10^6^ cells) compared to C57BL/6 mice (mean 1.6 × 10^6^ cells), consistent with the ELISA results, although the difference did not reach statistical significance (*p* = 0.09). As expected, no antigen-specific ASCs were detected in either strain of the control mice (Figure 1b, lower panel).

We also assessed the capacity of sera from Env-inoculated C57BL/6 mice to neutralize HIV-1 using a well-standardized Env pseudovirus assay and TZM-bl target cells [6]. We measured neutralizing activity against several Tier 1 clade B viruses, including HXBc2, MN, SF162 and BaL, with the highest neutralizing titers detected in mouse #2 against HXBc2 and MN (Table 1). No neutralizing Ab activity over background was detected for the more resistant 6535 virus and, importantly, no activity over a cut-off value of 20 was detected against the negative control virus pseudotyped with SIVmac Env. Overall the neutralizing Ab response was quite variable between the animals in the group. This highlights the need to develop higher resolution approaches to study neutralizing Ab responses in mice to understand the basis for the variability and to draw firm conclusions about the quality of the elicited response.

**Table 1:** HIV-1 neutralizing activity in sera of C57BL/6 mice 14 days after Env boosting.

Group	#ID	HXBc2	MN	SF162	BaL	6535	Neg Ctrl SIVmac
	**1**	97	15	20	49	14	18
	**2**	1151	768	47	10	13	10
**Immunized**	**3**	<5	58	38	30	5	<5
	**4**	17	24	21	28	19	7
	**5**	71	172	56	49	12	8
	**6**	27	32	16	30	5	9
							
**Adjuvant**	**1**	<5	<5	<5	8	<5	<5
**control**	**2**	<5	<5	5	13	5	16

### 3.2. Definition of Env-Specific Memory B Cells and GC B Cells in C57BL/6 Mice

**Figure 2:**
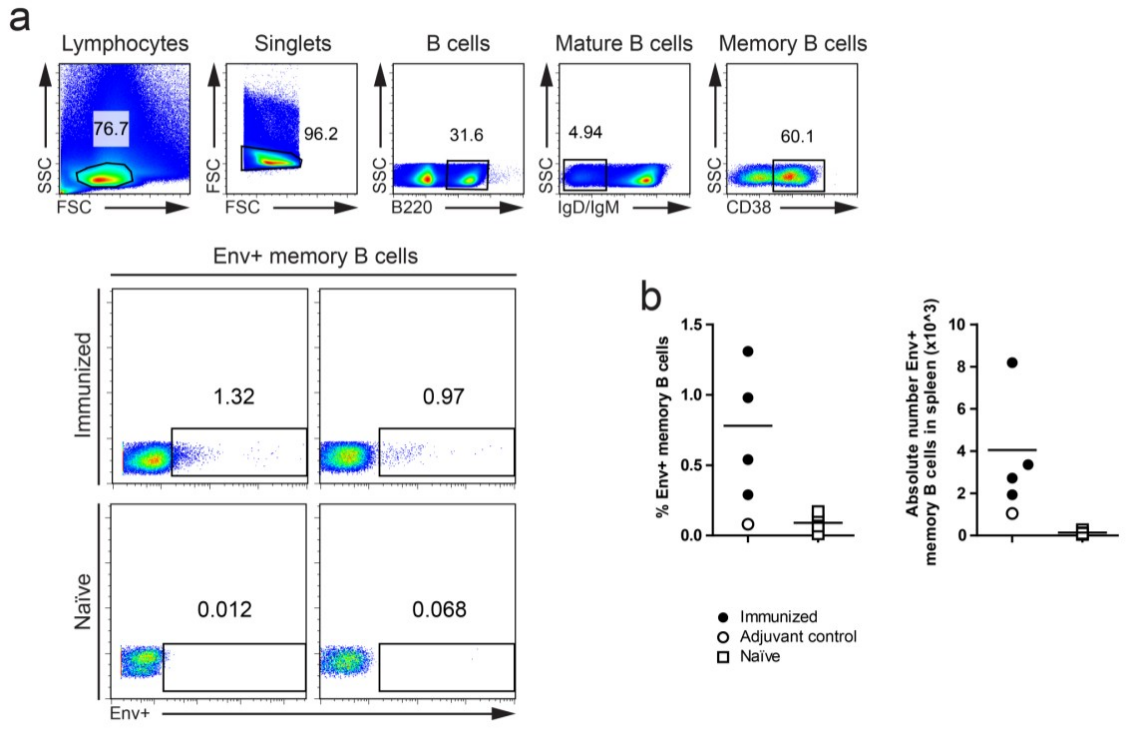
Env-specific memory B cells in the spleen of C57BL/6 mice after three s.c. inoculations with Env in AbISCO-100. The time interval between the first and second immunization was two weeks and between second and third immunization nine weeks. The data show memory B cells in spleen at day 6 after the last immunization. (**a**) Gating strategy for resolving Env-specific memory B cells. Memory B cells were defined as B220^+^, IgD/IgM^−^ CD38^+^ cells. Representative flow cytometry plots of cells stained with the Env probe from two immunized and two naïve control mice are shown. (**b**) Summary of individual mice. Percentage Env-specific memory B cells of total memory B cells, left panel, and absolute number of Env-specific memory B cells in the spleen, right panel. The results are from one experiment with four immunized mice, one adjuvant control mouse and four naïve mice.

One approach to increase the amount of information that can be obtained from studies of vaccine-induced B cell responses is to examine the response at the monoclonal level as a complement to analyses of polyclonal serum responses. In mice, the isolation of MAbs has traditionally been performed with hybridoma technologies, but recent advances in single cell RT-PCR of recombined Ab variable (V), diversity (D) and joining (J) gene segments from expressed mRNA of isolated B cells provide opportunities to clone Ab heavy chains (HC) and light chains (LC) for subsequent transfection and expression of full IgG molecules in mammalian cells [31]. We have successfully employed this approach to clone Env-specific memory B cells from HIV-1 Env-immunized rhesus macaques [8,10] and one of the objectives of the current study was to establish a similar protocol for mice. To optimize a gating strategy for Env-specific memory B cells we used mice immunized three times with Env and stained single mouse splenocytes for B220^+^, IgD/IgM^−^, CD38^+^ and Env^+^ cells as shown (Figure 2a). Env staining was achieved by using a biotinylated Env probe followed by streptavidin-APC. The percentage of Env^+^ memory B cells of total B220^+^, IgD/IgM^−^, CD38^+^ cells ranged between 0.3 and 1.4%, while the absolute cell numbers ranged from 2–8 × 10^3^ per spleen (Figure 2b). Very few Env-positive cells were detected from naïve or adjuvant only control animals suggesting that the background was low. We suggest that this protocol can be used for single cell sorting of Env-positive memory B cells for subsequent cloning and expression of the MAbs for verification of their specificities and characterization of their neutralizing properties.

**Figure 3:**
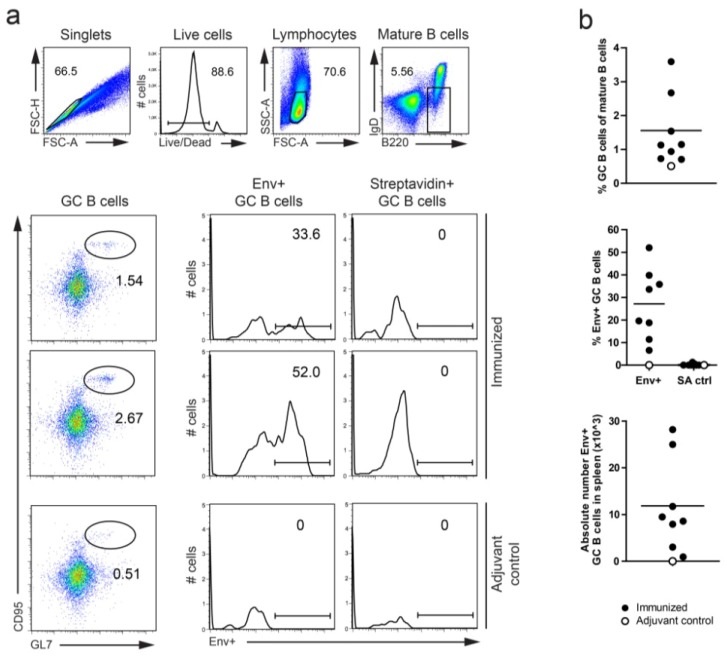
Detection of Env^+^ GC B cells in spleen 14 days after priming with Env and Alum Imject in C57BL/6 mice. (**a**) Gating strategy for measuring Env^+^ GC B cells in spleen. GC B cells were defined as B220^+^, IgD^−^, GL7^+^ and CD95^+^. GC B cells recognized by the Env biotinylated probe were considered Env^+^ GC B cells. As a negative control for each mouse, cells were stained with streptavidin-APC without the Env probe. Representative flow cytometry plots of cells stained with the Env probe from two immunized and one adjuvant control mouse are shown. (**b**) Summary of total GC B cell, Env^+^ GC B cell frequencies and absolute number of Env^+^ GC B cells in spleen of individual mice. The data are from one experiment with eight immunized mice and one adjuvant control mouse. Mean values are shown.

In addition to providing a strategy to study specificities archived in the memory B cell pool after prime-boosting, we wished to establish a protocol for the detection and isolation of Env-specific GC B cells after a single vaccine inoculation of C57BL/6 mice. To this end, we stained splenocytes after i.p. inoculation of Env administered to achieve maximal responses after a single immunization. In the first experiment, mice were sacrificed 14 days following Env inoculation and the splenocytes were resuspended and gated for the B220^+^, IgD^−^, CD95^+^ and GL7^+^ population to define the GC B cells. Similarly to the Env-specific memory B cell staining, we used the biotinylated Env probe followed by streptavidin-APC, while controls cells were stained with streptavidin-APC alone (Figure 3a). Env-positive GC B cells were observed in all Env-inoculated mice (n = 8), but not in the adjuvant control-immunized mouse. There was no staining with the streptavidin only control supporting the specificity of the Env probe. The animals varied in terms of the magnitude of the response, with the percentages GC B cells of mature B cells ranging from 0.5%–3.5% and absolute numbers of Env-specific B cells ranging from 1–30 × 10^3^ cells per spleen (Figure 3b). We did not consider this surprising given that the ELISA responses after priming generally are low. Nevertheless, the results are encouraging and illustrate that Env-specific GC B cells can be identified by flow cytometry for subsequent sorting and Ab cloning to define the ontogeny of the Env-specific B cell response.

## 4. Discussion

Antibodies define the correlate of protection for the majority of successful vaccines against infectious agents [32]. A critical step in the evaluation of vaccine candidates against HIV-1 is therefore the analysis of elicited neutralizing antibody responses. Given the challenges encountered by the HIV‑1 vaccine field so far, studies aimed at understanding the development of Env-specific humoral immune responses in greater details are critically needed. The development of robust protocols to study Env-specific B cell responses in mice would provide increased possibilities to investigate the limitations of current vaccine approaches and accelerate developments in the field.

To meet this goal, we first characterized, side-by-side, Ab responses elicited by subunit Env protein immunization of BALB/c and C57BL/6 mice. We observed that the magnitude of the Env-specific serum IgG antibody response was lower in C57BL/6 mice, which was also reflected as lower Env-specific IgG1, IgG2b and IgG3 concentrations in C57BL/6 mice compared to in BALB/c mice. As expected, BALB/c mice additionally produced Env-specific IgG2a antibodies, whilst C57BL/6 produced IgG2c. To further study the response we focused our subsequent efforts on C57BL/6 mice, the strain most frequently used in basic studies of B cell biology, with the aim to provide results that are generally applicable and of broad interest to the immunology field. We found that sera from Env‑inoculated C57BL/6 mice displayed neutralizing activity against several Tier 1 viruses, providing possibilities to study vaccine-induced neutralizing Abs in mice using similar methods as previously applied to non-human primates [11,33].

We further demonstrate that B cells capable of binding the gp140-F Env probe were detected by gating for B220^+^, IgD/IgM**^−^**, CD38^+^, Env^+^ cells (memory B cells) or B220^+^, IgD**^−^**, CD95^+^, GL7^+^, Env^+^ cells (GC B cells). In optimizing these protocols, we performed some kinetics experiments of the responses and found that Env-specific memory B cells are readily detected in the spleen 5 days after boosting (data not shown) but not at later time points. The GC response induced after priming peaked later, as expected from a primary response. Although a full kinetics analysis was not performed for Env, we chose to harvest spleens 14 days after priming when cell populations with phenotypic characteristics of GC B cells were observed in immunized mice with little background in adjuvant only inoculated control mice. Furthermore, while the characterization of neutralizing antibodies usually focuses on affinity‑matured IgG-switched B cells we opted to gate on the IgD/IgM**^−^** population rather than the IgG^+^ population in our staining protocol for Env-specific memory B cells. This decision was based on our finding that several of the commercially available (polyclonal) antibodies against mouse IgG are cross-reactive with IgD, excluding the possibility to use these anti-IgG reagents. We note that by staining for IgD/IgM**^−^** cells we cannot rule out the possibility that some of the cells are IgA^+^ or IgE^+^. However, by using primers that are specific for the mouse IgG Fc region in downstream PCR protocols it is still possible to preferentially isolate recombined V(D)J sequences from IgG-switched cells (data not shown). Similarly, we gated on IgD^−^ cells rather than IgG^+^ cells in the protocol for detection of GC B cells.

The frequencies of Env^+^ memory and GC B cells detected here are relatively low compared to frequencies reported from some studies of C57BL/6 mice inoculated with potent antigens such NP-protein conjugates [34,35,36]. This may be explained by a greater intrinsic immunogenicity of NP-protein conjugates in C57BL/6 mice, by use of other adjuvants and by the fact that a greater dose of antigen is used in most studies of NP-specific B cells, up to 50–100 µg protein per inoculation. In the current study we only used 10 µg soluble Env glycoprotein per inoculation since the gp140-F immunogen is not commercially available but is produced in our laboratory by transient transfection of 293F cells using a previously described protocol [20]. Thus, by increasing the dose of antigen, and/or using a stronger adjuvant, it is likely that increased numbers of Env^+^ can be detected. Nevertheless, even with the conditions used here we show that it is possible to isolate Env^+^ cells for subsequent Ab repertoire analysis and MAb cloning.

In summary, we have established protocols to identify Env-specific memory B cells or GC B cells from Env immunized C57BL/6 mice. Examination of GC B cells isolated after primary immunization can provide direct information about the specificities of initial B cell clones that have entered GCs while isolation of memory B cells provides information about how the response persists and evolves over time, information that can guide the design of improved immunization regimens [33]. Recently, several approaches to enhance the immunogenicity of recombinant protein-based subunit vaccines were proposed [37,38,39,40,41,42] and it would be of interest to evaluate their impact on HIV-1 Env‑elicited B cell responses at the single cell level. We show that, while responses to subunit HIV-1 Env are low, memory and GC B cells can be detected after immunization for subsequent analysis of the elicited response at a greater level of detail.
